# Substance P Receptor Signaling Mediates Doxorubicin-Induced Cardiomyocyte Apoptosis and Triple-Negative Breast Cancer Chemoresistance

**DOI:** 10.1155/2016/1959270

**Published:** 2016-02-11

**Authors:** Prema Robinson, Moses Kasembeli, Uddalak Bharadwaj, Nikita Engineer, Kris T. Eckols, David J. Tweardy

**Affiliations:** ^1^Department of Infectious Diseases, Infection Control, and Employee Health, The University of Texas MD Anderson Cancer Center, Houston, TX 77030, USA; ^2^Department of Natural Science and Mathematics, Lee University, Cleveland, TN 37311, USA; ^3^Department of Molecular and Cellular Oncology, The University of Texas MD Anderson Cancer Center, Houston, TX 77030, USA

## Abstract

Doxorubicin (DOX), an anthracycline, is broadly considered the most active single agent available for treating breast cancer but has been known to induce cardiotoxicity. Although DOX is highly effective in treating triple-negative breast cancer (TNBC), DOX can have poor outcomes owing to induction of chemoresistance. There is an urgent need to develop new therapies for TNBC aimed at improving DOX outcome and DOX-induced cardiotoxicity. Substance P (SP), a neuropeptide involved in pain transmission is known to stimulate production of reactive oxygen species (ROS). Elevated cardiac ROS is linked with heart injury and failure. We investigated the role of SP in chemotherapy-associated death of cardiomyocytes and chemoresistance. We showed that pretreating a cardiomyocyte cell line (H9C2) and a TNBC cell line (MDA-MB 231) with aprepitant, a SP receptor antagonist that is routinely used to treat chemotherapy-associated associated nausea, decreased DOX-induced reduction of cell viability, apoptotic cell death, and ROS production in cardiomyocytes and increased DOX-induced reduction of cell viability, apoptotic cell death, and ROS production in TNBC cells compared with cells treated with DOX alone. Our findings demonstrate the ability of aprepitant to decrease DOX-induced killing of cardiomyocytes and to increase cancer cell sensitivity to DOX, which has tremendous clinical significance.

## 1. Introduction

Doxorubicin (DOX), an anthracycline, is broadly considered the most active single agent available for treating breast cancer [1–3]. However, DOX has been known to induce cardiotoxic side effects such as electrocardiographic changes, decreased left ventricular ejection fraction values, and life-threatening heart failure or acute coronary syndromes in some patients [4–6]. There is an urgent need to develop new noncardiotoxic therapies. Importantly, DOX often also induces chemoresistance. For example, though DOX is highly effective in treating triple-negative breast cancer (TNBC; lacking 3 of the hormone/molecular receptors/markers, ER2, PR2, Her-22), DOX can have poor outcomes owing to induction of chemoresistance [7, 8]. Breast cancer is one of the leading causes of cancer-associated death in women. In the United States alone, each year, more than 260,000 new cases of breast cancer are diagnosed, and more than 40,000 breast cancer-associated deaths occur [9, 10]. Given the lack of validated molecular targets and the poor outcomes in TNBC, there is an urgent need for new therapies to prevent chemoresistance.

Substance P (SP), an 11-amino acid neuropeptide involved in pain transmission, is made by and elicits response from nerves, endothelial cells, and cells of the immune system [11–17]. SP mediates pain, neurogenic inflammation, and mitogenesis via interaction with its high-affinity receptor NK-1R, which is widely distributed throughout the body. SP stimulates production of reactive oxygen species (ROS) [18, 19]. Elevated cardiac ROS is linked with heart, injury, and failure in other cardiac settings [20, 21]. We have previously demonstrated that SP was elevated in hearts of mice infected with the encephalomyocarditis virus, which causes viral myocarditis [22]. We also have previously shown that, in mice infected with a parasite that causes cysticercosis and in those infected with encephalomyocarditis virus, the mortality, heart weight to body weight ratios, cardiomyocyte, hypertrophy, and apoptosis were significantly higher than that of uninfected mice. In contrast, SP-deficient mice and/or NK-1R antagonist-treated animals were protected against all these effects [22]. Other groups have shown that SP is involved in inducing chronic volume overload-induced heart failure and that deletion of the SP gene protected mice from developing left ventricular hypertrophy in the form of ventricular dilatation [23]. Furthermore, studies have shown that animals with magnesium deficiency had higher SP levels in their cardiac lesions than did normal animals [24] and that blockade of NK-1R significantly reduced ROS production in cardiac cells and improved diastolic and systolic function in these animals [24, 25]. These findings suggest that elevated SP can be detrimental to the heart and that NK-1R antagonism can be used to treat SP-induced cardiac manifestations.

SP and NK-1R have been detected in tumor cells and in intra- and peritumoral blood vessels [26–28]; furthermore, SP has been shown to protect tumor cells from apoptosis [29]. The relevance of the SP/NK-1 receptor system has been specifically shown in pancreatic cancer, where SP is involved in pancreatic cancer proliferation, neoangiogenesis, and migration of pancreatic cancer cells and SP receptor antagonism has been shown to reverse these alterations [26, 29–31]. These findings suggest that elevated SP can be detrimental in cancer and suggest that NK-1R antagonism can be beneficial in cancer treatment.

We hypothesized that SP plays a role in chemotherapy-associated death of cardiomyocytes and in chemoresistance. In order to test this hypothesis, we determined the effects of aprepitant, an antagonist of SP receptor (neurokinin 1 receptor [NK-1R]) that is routinely used to treat chemotherapy-associated nausea, on DOX-induced reduction of cell viability, apoptotic cell death, and ROS production by cardiomyocytes and TNBC cells. We showed that pretreating a cardiomyocyte cell line (H9C2) and a TNBC cell line (MDA-MB 231) with aprepitant decreased DOX-induced reduction of cell viability in the cardiomyocytes and increased DOX-induced reduction of cell viability in TNBC cells compared to cells treated with DOX alone. Also, the levels of apoptotic cell death and ROS in response to DOX were decreased in aprepitant pretreated cardiomyocyte cells and were increased in aprepitant pretreated TNBC cells compared to the untreated cells.

Currently, no studies have investigated SP as a mediator of chemotherapy-induced cardiotoxicity or the role of SP antagonism as a synergistic mechanism to enhance chemotherapy's ability to kill resistant TNBC cells. These studies show that SP plays a dual detrimental role in DOX-associated killing of cardiomyocytes and induction of chemoresistance in TNBC and has implications for tremendous future clinical translational relevance. These studies may lead to use of SP receptor antagonism, for prevention of DOX-mediated toxicity and at the same time for increment of antitumor effects of DOX for TNBC and probably other cancers.

## 2. Material and Methods

### 2.1. Cell Culture and Chemicals

Rat cardiomyocyte cell line, H9C2, and human TNBC breast cancer cell line, MDA-MB-231, were purchased from the American Type Culture Collection (Manassas, VA). Culture media, antibiotics, fetal bovine serum, and other supplements were bought from Invitrogen (Carlsbad, CA). Cells were maintained in complete media with 10% fetal bovine serum, antibiotics (streptomycin and penicillin), an antifungal agent (amphotericin B), GlutaMAX (Thermo Fisher Scientific, Waltham, MA), and pyruvate and were not passed continuously more than 4 weeks. Doxorubicin and aprepitant (resp., 15007 and 4867) were purchased from Cayman Chemicals, Ann Arbor, Michigan.

### 2.2. MTT Assay

To determine the effect of aprepitant on cell proliferation, we measured cell viability using the MTT assay. Following treatment with the indicated concentrations of DOX with and without aprepitant or with aprepitant or media or vehicle alone, cells were dispersed by trypsinization and seeded at 8,000–10,000 cells/well in a 96-well plate overnight before being treated. Subsequently, MTT (1 mg/mL) in medium with 1% serum was added to each well, and the wells were incubated for 2 h at 37°C. An extraction buffer (20% sodium dodecyl sulfate and 50% dimethylformamide) was added, and the cells were incubated overnight at 37°C. The optical density was measured at 590 nm using a 96-well multiscanner (Molecular Devices, Sunnyvale, CA). The proliferating capacity of the cell was measured by dividing the viability at a certain experimental condition by the viability of corresponding controls (media or vehicle control). Data are presented as percentage viability related to untreated cells ± SEM for each group.

### 2.3. TUNEL Assay

The effect of aprepitant on cell death was determined by measuring levels of apoptotic cells using the TUNEL assay. The ApopTag Plus Peroxidase In Situ Apoptosis Detection Kit (EMD Millipore, Billerica, MA) was used to detect apoptotic cells, according to the manufacturer's instructions [32]. Briefly, following treatment with the indicated concentrations of DOX with and without aprepitant or with aprepitant or media or vehicle alone, cells on tissue culture chamber slides were fixed in 1% paraformaldehyde in phosphate-buffered saline solution (PBS, pH 7.4) for 10 min at room temperature, followed by 2 washes with 1x PBS for 5 min each wash. Samples were postfixed in precooled ethanol : acetic acid (2 : 1) for 5 min at −20°C to subject the cells to permeabilization. Cells were then quenched in 3.0% hydrogen peroxide in PBS for 5 min at room temperature, were rinsed twice with PBS or distilled water for 5 min each time, and were treated with equilibration buffer for 1 min. The equilibration buffer was then drained, and the cells were treated with terminal deoxynucleotidyl transferase enzyme for 1 h at 37°C. The cells were then treated with stop-wash buffer and were washed with PBS 3 times (1 min each wash) followed by treatment with antidigoxigenin conjugate (30 min, room temperature (RT)), 1 wash with PBS (1 min, RT), treatment with peroxidase substrate (3–6 min, RT), and 3 washes with distilled water (1 min each). The samples were counterstained with hematoxylin, were mounted, and were viewed under a light microscope. The number of TUNEL-positive nuclei was counted in 10 randomly chosen high-power fields (400x) of each slide by an experienced microscopist blinded to the study design. The percentage of positive cells was calculated. Data are presented as percentage of positive cells ± SEM for each group.

### 2.4. ROS Measurement

The effect of aprepitant on ROS production was determined by measuring ROS levels by the dichlorofluorescein diacetate (DCFDA) assay. Following treatment with the indicated concentrations of DOX with and without aprepitant or with aprepitant or media or vehicle alone, cells were stained with DCFDA (5 *μ*M) for 30 min at 37°C in the dark. ROS production was determined by fluorescence spectroscopy with maximum excitation and emission spectra of 495 nm and 529 nm, respectively. Data are presented as fluorescence intensity ± SEM for each group.

### 2.5. Quantitation of SP Protein

To determine whether DOX treatment increased SP levels in cardiomyocytes and cancer cells, we treated H9C2 and MDA-MB 231 cells with their respective median inhibitory concentration (IC_50_) doses of DOX and then determined SP levels in cell lysates. Quantitation of SP protein was performed as described previously [33]. Briefly, cells with and without DOX treatment were washed once and then reconstituted with cold 1x PBS containing protease inhibitor cocktail (04 693 132 001, Roche, Indianapolis, IN). Cells were scraped, were spun at 1500 rpm for 10 min, were reconstituted in lysis buffer (43-040, Cell Signaling, Danvers, MA), and were incubated on ice for 15 min. The lysed cells were then spun at 12,000 rpm for 15 min, and the supernatant was used for SP quantitation by using an enzyme-linked immunosorbent assay kit from Enzo Life Sciences (ADI-900-018, Farmingdale, NY). Total protein was quantified using the Bradford method (500-0006, Bio-Rad, Hercules, CA). Results are expressed as picogram of SP per milligram of total protein ± SEM for each group.

### 2.6. Statistical Analyses

Data presented are mean ± SEM of a minimum of 2 experiments. Statistical differences were determined using analysis of variance (ANOVA), followed by Tukey's or Dunn's posttest as appropriate or by Student's unpaired *t*-test. Statistical significance was set at *p* ≤ 0.05. Data and statistical analysis were performed using Graph Pad Prism version 6.04 for Windows, Graph Pad Software (San Diego, CA).

## 3. Results

### 3.1. SP Receptor Antagonism Prevents DOX-Induced Reduction in Cardiomyocyte Viability

**Figure 1:**
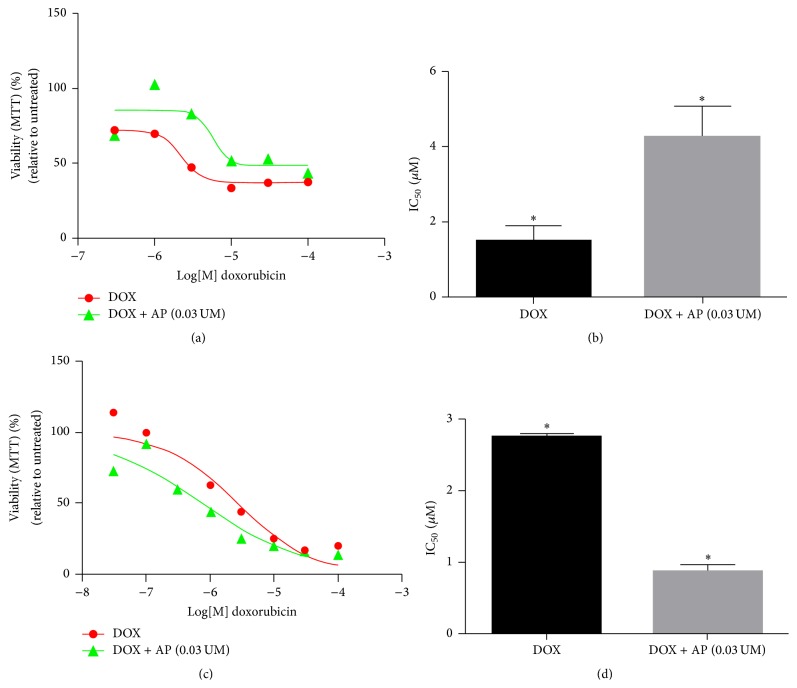
Effect of substance P receptor antagonist pretreatment on doxorubicin-induced cardiomyocyte growth inhibition and triple-negative breast cancer cell chemoresistance. Levels of viability as determined by the MTT assay, in response to DOX in aprepitant pretreated versus untreated rat H9C2 cardiomyocytes (a and b) and MDA-MB 231 TNBC cells (c and d) (^*∗*^ *p* ≤ 0.05, ANOVA, *n* = 2, for both).

In order to determine if SP receptor antagonism prevents DOX-induced reduction in cardiomyocyte viability, H9C2 cells were seeded in a 96-well plate (10,000 cells per well); after 24 hrs, duplicate wells were treated with different concentrations of DOX (ranging from 0.03 *μ*m to 100 *μ*m) with and without aprepitant pretreatment (0.03 *μ*m, 2 hrs before DOX treatment). Control wells included treatment with the corresponding concentrations of vehicle (DMSO) used for reconstituting the aprepitant (0.0005% DMSO in water). Also included was a group of wells treated with aprepitant alone. All experiments were performed at least twice and results are expressed as mean ± SEM, unless otherwise indicated. We determined that aprepitant pretreatment decreased the DOX-induced loss of cell viability compared with DOX alone. The median inhibitory concentration (IC_50_) of DOX was 1.46 *μ*m ± 0.4 *μ*m; aprepitant pretreatment led to a 3-fold increase in the IC_50_ levels to 4.23 *μ*m ± 0.8 *μ*m (Figures 1(a) and 1(b); *p* < 0.05, ANOVA, *n* = 2).

### 3.2. SP Receptor Antagonism Reverses Chemoresistance of MDA-MB 231 TNBC Cells

To determine if SP receptor antagonism is beneficial in decreasing chemoresistance of TNBC cells, we determined whether aprepitant is beneficial in increasing the DOX-mediated killing of MDA-MB 231 TNBC cells. DOX and aprepitant treatment were as above. All results are expressed as mean ± SEM, unless otherwise indicated. We determined that aprepitant pretreatment increased the DOX-induced killing compared with DOX alone. The IC_50_ of DOX was 2.74 *μ*m ± 0.05 *μ*m; aprepitant pretreatment led to a 3.14-fold decrease in the IC_50_ levels to 0.87 *μ*m ± 0.10 *μ*m (Figures 1(c) and 1(d); *p* < 0.05, ANOVA, *n* = 2).

### 3.3. SP Receptor Antagonism Led to Decreased Levels of Apoptosis of H9C2 Cardiomyocytes

**Figure 2:**
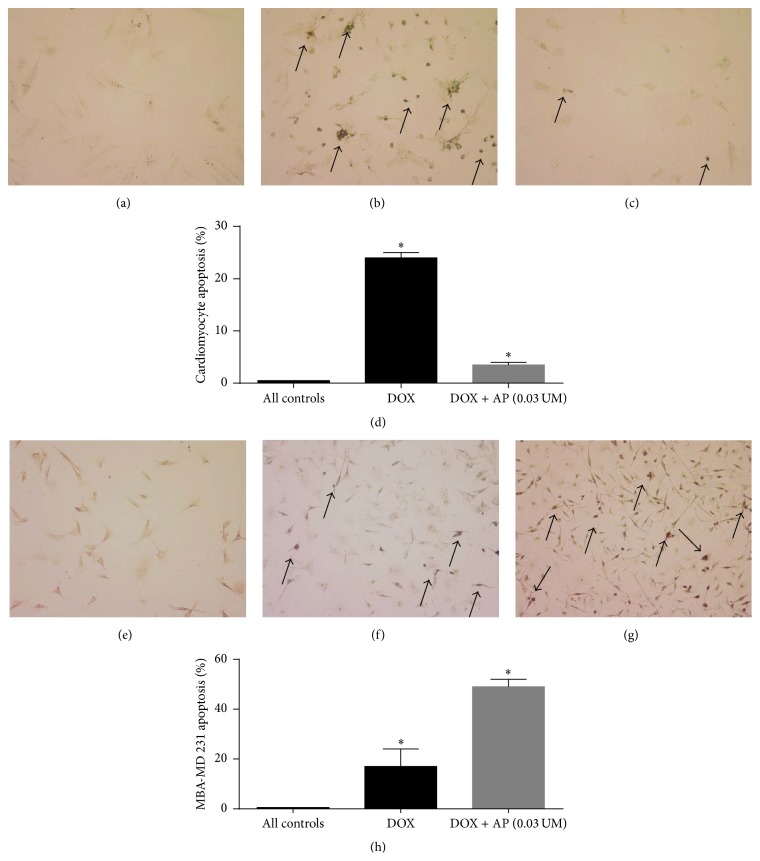
Effect of substance P receptor antagonist pretreatment on doxorubicin-induced apoptosis of cardiomyocytes and triple-negative breast cancer cells. Levels of apoptosis as determined by the TUNEL assay, in response to DOX in aprepitant pretreated versus untreated rat H9C2 cardiomyocytes and MDA-MB 231 TNBC cells. A photomicrograph of H9C2 cells from (a) control aprepitant treated, (b) DOX-treated, and (c) DOX + aprepitant pretreated cells showing numerous strongly positive brown apoptotic nuclei in the DOX group and very few faintly positive nuclei in the DOX + aprepitant group (arrows depict positive cells, original magnification 200x). (d) Number of apoptotic H9C2 cells in the 2 experimental groups and all control groups (media, vehicle, and aprepitant alone groups, all showing no positive apoptotic cells) (^*∗*^ *p* ≤ 0.05, ANOVA, *n* = 2). Only statistical comparisons between DOX and DOX + AP are shown. A photomicrograph of MBA-MD 231 cells from (e) control aprepitant treated, (f) DOX-treated, and (g) DOX + aprepitant pretreated cells showing numerous strongly positive brown apoptotic nuclei in the DOX + aprepitant group versus the group treated with DOX alone (arrows depict positive cells, original magnification 200x). (h) Number of apoptotic MBA-MD 231 cells in the 2 experimental groups and all control groups (media, vehicle, and aprepitant alone groups, all showing no positive apoptotic cells) (^*∗*^ *p* ≤ 0.05, ANOVA, *n* = 2). Only statistical comparisons between DOX and DOX + AP are shown.

To determine whether the protective effects of SP antagonist pretreatment on reduction of DOX-induced reduction of viability of cardiomyocytes was accompanied by decreased levels of apoptosis, we determined the levels of apoptotic cells in response to DOX in aprepitant pretreated versus untreated H9C2 cardiomyocytes. We determined that aprepitant pretreatment reduced the DOX-induced level of apoptotic TUNEL-positive cardiomyocytes by 7-fold compared to DOX alone (Figures 2(a)–2(c)). The percentage of positive apoptotic cells in the DOX alone group was 24% ± 1%; aprepitant pretreatment reduced the percentage of positive apoptotic cells to 3.5% ± 0.5% (Figure 2(d), *p* < 0.05, ANOVA, *n* = 2). Control groups (media, vehicle, and aprepitant alone groups) did not have any positive apoptotic cells.

### 3.4. SP Receptor Antagonism Led to Increased Levels of Apoptosis of MDA-MB 231 TNBC Cells

To determine whether the beneficial effects of SP antagonist pretreatment on increment of DOX-induced reduction of viability of MDA-MB 231 TNBC cells were accompanied by increased levels of apoptosis, we determined the levels of apoptotic cells in response to DOX in aprepitant pretreated versus untreated MDA-MB 231 TNBC cells. We determined that aprepitant pretreatment increased the DOX-induced level of apoptotic TUNEL-positive cells by 3-fold compared to DOX alone (Figures 2(e)–2(g)). The percentage of positive apoptotic cells in the DOX alone group was 17%  ±  7%; aprepitant pretreatment increased the percentage of positive apoptotic cells to 49 ± 3 (Figure 2(h), *p* < 0.05, ANOVA, *n* = 2). Control groups (media, vehicle, and aprepitant alone groups) did not have any positive apoptotic cells.

### 3.5. SP Receptor Antagonism Inhibits DOX-Induced ROS Production by H9C2 Cardiomyocytes

**Figure 3:**
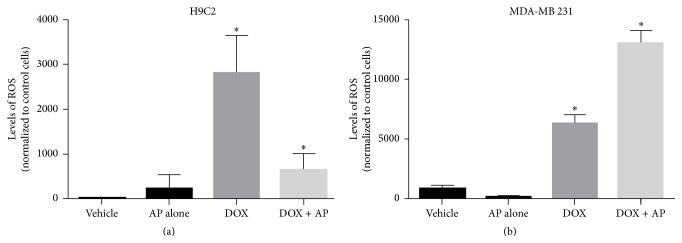
Effect of substance P receptor antagonist pretreatment on doxorubicin-induced, reactive oxygen species (ROS) production in cardiomyocytes and triple-negative breast cancer cells. Levels of ROS as determined by the DCFDA assay, in response to DOX in aprepitant pretreated versus untreated rat H9C2 cardiomyocytes (a) and MDA-MB 231 TNBC cells (b) (^*∗*^ *p* ≤ 0.05, ANOVA, *n* = 2, for both). Only statistical comparisons between DOX and DOX + AP are shown.

To determine whether the protective effects of SP antagonism on DOX-induced killing of cardiomyocytes were accompanied by decreased ROS levels, we determined the levels of ROS in response to DOX in pretreated versus untreated H9C2 cardiomyocytes. We determined that aprepitant pretreatment decreased DOX-induced ROS production compared with DOX alone. The level of ROS as seen by fluorescence intensity produced by 24,000 cells in response to DOX alone was 2804 ± 601.5 units, whereas aprepitant pretreatment led to a 4.3-fold decrease in levels of ROS to 651.5 ± 259.5 units (Figure 3(a), *p* < 0.05, ANOVA, *n* = 2).

### 3.6. SP Receptor Antagonism Led to Increased Levels of ROS in Response to DOX in MDA-MB 231 TNBC Cells

To determine whether SP antagonist pretreatment induced increased DOX-induced killing of TNBC's was accompanied by increased ROS levels, we determined the levels of ROS in response to DOX in aprepitant pretreated versus untreated MDA-MB 231 TNBC cells. We determined that aprepitant pretreatment increased DOX-induced ROS production in the MDA-MB 231 TNBC cells compared with DOX alone. The level of ROS as seen by fluorescence intensity produced by 24000 cells in response to DOX alone was 5856 ± 372 units, whereas aprepitant pretreatment led to a 2.7-fold increase in levels of ROS to 13828 ± 137.5 units (Figure 3(b), *p* < 0.05, ANOVA, *n* = 2).

### 3.7. DOX Increases SP Levels in Both H9C2 Cardiomyocytes and MDA-MB 231 TNBC Cells

**Figure 4:**
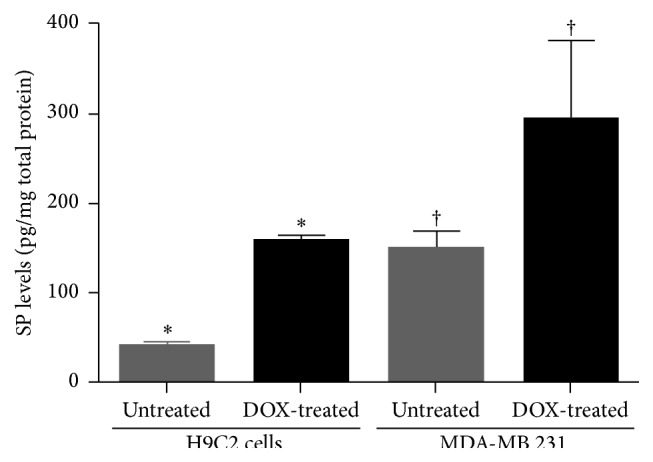
Levels of SP are increased in response to DOX in cardiomyocytes and TNBC cells. Levels of SP as determined by ELISA, in H9C2 and MDA-MB 231 cells with and without DOX treatment (^*∗*, †^ *p* ≤ 0.05, *t*-test, *n* = 2).

To confirm that aprepitant-mediated effects were mediated via SP, we investigated whether DOX treatment increased SP levels in cardiomyocytes and cancer cells. We treated H9C2 and MDA-MB 231 cells with their respective IC_50_ dose of DOX (i.e., 1.5 *μ*m for H9C2 and 2.74 *μ*m for MDA-MB 231 cells) and then determined SP levels by ELISA in the cell lysates. We determined that DOX-treated H9C2 cells and MDA-MB 231 cells, respectively, had a 2.2-fold and a 4-fold increase in the level of SP compared to that of untreated cells (H9C2: DOX-treated, 156 ± 7 pg; untreated, 39 ± 5 pg; *p* ≤ 0.05) (MDA-MB 231: DOX-treated, 293 ± 88 pg; untreated, 149 ± 19 pg, *p* ≤ 0.05, *t*-test for both, *n* = 2 for both, Figure 4).

## 4. Discussion

Breast cancer is one of the leading causes of cancer-associated death in women [2]. Although DOX has shown to be highly effective in treating TNBC, it has a poor outcome owing to induction of resistance. Most importantly, DOX is associated with induction of cardiotoxicity in many patients. There is an urgent need to develop new noncardiotoxic therapies for cancer or prevent DOX-mediated toxicity without reducing its antitumor effects.

In the current paper, we determined the role of SP in chemotherapy-associated death of cardiomyocytes and chemoresistance of TNBC cells. We showed that pretreating a cardiomyocyte cell line (H9C2) and a TNBC cell line (MDA-MB 231) with aprepitant, a SP receptor antagonist that is routinely used to treat chemotherapy-associated nausea, decreased DOX-induced reduction of viability, apoptotic cell death, and ROS production in cardiomyocytes and increased DOX-induced reduction of viability, apoptotic cell death, and ROS production in TNBC cells compared with cells treated with DOX alone. These studies show that SP plays a dual detrimental role in induction of DOX-associated killing of cardiomyocytes and induction of chemoresistance in TNBC.

We do not know the mechanism by which SP enhances chemotherapy-associated killing of cardiomyocytes or induces chemoresistance. DOX is known to induce cardiotoxicity via induction of DNA double-strand breaks. Studies have shown that inhibiting the enzyme topoisomerase IIB (TOPIIB), which is responsible for unwinding supercoiled DNA strands during replication, prevents DOX-induced cardiotoxicity [34]. We speculate that the mechanism by which SP mediates DOX-induced cardiotoxicity may be via the SP/NK-1R pathway-associated molecules Rac1 (Ras-related C3 botulinum toxin substrate 1) and Nur77 (nerve growth factor IB). Both molecules are associated with apoptotic cell death linked to TOPIIB [35] and/or nonapoptotic cell death [32, 36–39]. We speculate that the mechanism by which SP mediates DOX-induced chemoresistance in TNBC may be via activation of Forkhead box protein M1 (FOXM1, a transcription factor that is linked to increased survival of tumor cells) and programmed cell death 1 (PD-1, an immune surveillance escape factor) [33, 40, 41]. In future studies, we aim to determine the exact mechanisms involved in both SP-induced events. As part of future studies, we will determine levels of TOPIIB, Rac1, and Nur77 in H9C2 cells and levels of FOXM1 and PD-1 in TNBC cells, treated with and without DOX and/or aprepitant. Also, as part of our future studies we will determine whether a new synergistic therapy consisting of aprepitant + DOX will prevent chemotherapy-associated cardiotoxicity and improve the outcome of TNBC using in vivo murine models of cardiotoxicity and TNBC.

Although ours and other studies using murine viral, parasitic, and other heart failure models have shown that elevated SP can be detrimental to the heart and can cause cardiac manifestations such as dilated cardiomyopathy and chronic volume overload-induced heart failure [22–25], there are no studies that demonstrate increased cardiac SP and its high-affinity receptor, NK1, in malignancy or doxorubicin therapy. Most importantly, currently no studies have investigated SP as a mediator of chemotherapy-induced cardiotoxicity.

Similarly, other studies using murine, pancreatic cancer models, human breast cancer tissues, and in vitro pancreatic cell lines and TNBC cell lines have shown elevated SP and or NK-1R expression, linked to proliferation, migration, and cancer metastases [26–31, 42–48]. Furthermore, very important studies by Dr. Munoz's group have shown that SP receptor antagonist aprepitant has antitumor action against breast cancer cell lines and also that doxorubicin has synergic effect with aprepitant against human hepatoblastoma cell lines [48, 49]. Although the above studies demonstrate the importance of SP/NK-1R pathway in cancers, and also demonstrate the role of SP antagonism as a synergistic therapy with DOX in hepatoblastoma cells, there are no studies that have investigated the role of SP antagonism as a synergistic mechanism to enhance chemotherapy's ability to kill resistant TNBC cells. Most importantly, there are no studies that have investigated if SP receptor antagonism will play a dual protective role to prevent chemoresistance of TNBC and at the same time prevent cardiotoxicity. Our findings show that SP plays a dual detrimental role in induction of DOX-associated killing of cardiomyocytes and induction of chemoresistance in TNBC. These studies, showing SP receptor antagonism to decrease DOX-induced killing of cardiomyocytes and to increase cancer cell sensitivity to DOX, have the potential for development into tremendous future clinical translation. These studies may lead to use of SP receptor antagonism, for prevention of DOX-mediated cardiotoxicity and enhancement of antitumor effects of DOX for TNBC and probably other cancers.
